# Specific expression of lacZ and cre recombinase in fetal thymic epithelial cells by multiplex gene targeting at the *Foxn1 *locus

**DOI:** 10.1186/1471-213X-7-69

**Published:** 2007-06-18

**Authors:** Julie Gordon, Shiyun Xiao, Bernard Hughes, Dong-ming Su, Samuel P Navarre, Brian G Condie, Nancy R Manley

**Affiliations:** 1 Department of Genetics, University of Georgia, Athens, GA 30602, USA; 2 Dept. of Biomedical Research, The University of Texas Health Center at Tyler, 11937 US Highway 271, Tyler, TX 75708, USA; 3 Institute for Molecular Medicine and Genetics, Medical College of Georgia, Augusta, GA 30912, USA

## Abstract

**Background:**

Thymic epithelial cells (TECs) promote thymocyte maturation and are required for the early stages of thymocyte development and for positive selection. However, investigation of the mechanisms by which TECs perform these functions has been inhibited by the lack of genetic tools. Since the *Foxn1 *gene is expressed in all presumptive TECs from the early stages of thymus organogenesis and broadly in the adult thymus, it is an ideal locus for driving gene expression in differentiating and mature TECs.

**Results:**

We generated two knock-in alleles of Foxn1 by inserting IRES-Cre or IRES-lacZ cassettes into the 3' UTR of the *Foxn1 *locus. We simultaneously electroporated the two targeting vectors to generate the two independent alleles in the same experiment, demonstrating the feasibility of multiplex gene targeting at this locus. Our analysis shows that the knockin alleles drive expression of Cre or lacZ in all TECs in the fetal thymus. Furthermore, the knockin alleles express Cre or lacZ in a *Foxn1*-like pattern without disrupting *Foxn1 *function as determined by phenotype analysis of *Foxn1 *knockin/*Foxn1 *null compound heterozygotes.

**Conclusion:**

These data show that multiplex gene targeting into the 3' UTR of the *Foxn1 *locus is an efficient method to express any gene of interest in TECs from the earliest stage of thymus organogenesis. The resulting alleles will make possible new molecular and genetic studies of TEC differentiation and function. We also discuss evidence indicating that gene targeting into the 3' UTR is a technique that may be broadly applicable for the generation of genetically neutral driver strains.

## Background

Thymic epithelial cells (TECs) perform an essential function to promote many aspects of T cell maturation within the thymus, including thymocyte proliferation, apoptosis, and positive and negative selection [1,2]. However, there are still many gaps in our knowledge of the molecular mechanisms operating within TECs to control these diverse functions. A major obstacle has been the lack of genetic tools for manipulating gene expression specifically in TECs, which has hampered analysis of these molecular mechanisms. While keratin promoters can drive expression of transgenes in subsets or all of TECs [3], they are also expressed widely in epithelium, which restricts their utility for analysis of thymus phenotypes. To circumvent this problem, a recent study made use of embryo chimeras using nude mouse donors and homozygous knockout embryonic stem cells (ES cells) [4]; however, this technique is technically challenging, time consuming, and is limited by the availability of homozygous knockout ES cells. Identifying an efficient and reproducible genetic method for expressing genes in TECs and generating TEC-specific gene knockouts would enable new molecular and genetic studies of TEC differentiation and function.

The *Foxn1 *gene is expressed in all epithelial cells in the early thymic rudiment from E11.5 [5,6], and is required cell-autonomously for TEC differentiation [7]. The *Foxn1 *null allele, nude, has a complete failure of TEC differentiation. Outside of the thymus, *Foxn1 *has a very restricted expression pattern, limited to developing hair follicles and skin [8,6,9]. The first targeted allele of *Foxn1 *contained an IRES-lacZ insertion into the third exon, creating a tagged null allele which was used to show expression of *Foxn1 *in both cortical and medullary TECs [6], and to identify the initial expression pattern of *Foxn1 *during thymic ontogeny [5]. Thus, the *Foxn1 *gene is a good locus for expressing genes in the thymic epithelium from very early stages.

Gene targeting in embryonic stem cells is commonly used to generate alleles of genes tagged with marker genes (β-galactosidase/lacZ, hPAP, fluorescent proteins), or to express other genes of interest such as Cre recombinase under the control of an endogenous promoter. Loci that are designed to express exogenous sequences under the control of a gene are often referred to as "driver" loci. This approach generally combines creation of a mutant allele with the insertion of a sequence to be expressed. The downside of such a "knockin-knockout" approach occurs when the driver alleles are combined with mutations in other genes. For example, the use of a knockin/knockout Cre driver locus in a conditional knockout strategy may result in additional phenotypes due simply to the genetic interaction between the Cre driver and the gene of interest.

In addition, there are many loci for which any disruption of function will lead to a phenotypic effect (*Pax6*, *Pax1*, *Tbx1*, *Sox9*, to name a few). Many of these loci are expressed in temporal and spatial patterns that make them attractive for use as driver loci for the analysis of development. The haploinsufficiency of these loci can in theory be bypassed by the use of promoter fragments or BAC-based transgenes derived from these loci to express Cre from randomly integrated transgenes. While this approach can certainly be successful, it also can have significant well-known problems. These include the lack of identified regulatory elements for many genes, the potential for widely distributed regulatory elements, the need to screen multiple lines to identify correct expression relative to the endogenous gene, instability of expression patterns for a given line over time, and mutation of genes at the insertion site. A recently published *Foxn1::EGFP *transgenic line is a good case in point [10]. Although this transgene does have correct early fetal expression and is expressed in the adult thymus, its expression relative to the endogenous gene also must be validated independently relative to the endogenous gene at all stages, to verify that all of the required regulatory sequences for correct expression through the life of the animal are present, and that it accurately reflects endogenous gene expression. Thus, there are distinct disadvantages to this approach, particularly as a widely applicable strategy.

The creation of multiple different tagged alleles is also extremely useful for multiple mutant analyses. As the number of lacZ and GFP-tagged alleles for genes increases, the ability to generate a "rainbow" series of alleles at key loci for marker analyses in mutants becomes increasingly valuable. However, the generation of multiple alleles of the same gene is expensive, time consuming, and labor-intensive, resulting in a practical barrier to the generation of these useful allelic series.

The *Foxn1 *genomic locus has been shown by us and others to undergo very high efficiency gene targeting in ES cells, with frequencies up to 95% of correctly targeted loci using positive-negative selection [6,11]. We used an approach that we have termed multiplex gene targeting, which allows the simultaneous generation of multiple alleles for a given gene in a single gene targeting experiment. The general approach is to generate targeting vectors that are identical in all respects, except for the sequence of the inserted cassette. Correctly targeted cell lines for each vector can then be generated in a single experiment simply by electroporating a mixture of the vectors and then screening for the two alleles. In the current example two targeting vectors were generated, each containing a different marker (Cre recombinase or lacZ) with an internal ribosomal entry sequence (IRES) inserted at the same location in the *Foxn1 *3' untranslated region (UTR). The targeting events in the resulting ES cell lines resulted in the expected allele structure, generating multiple independent ES cell lines for either the lacZ or Cre alleles in a single experiment. These results demonstrate that multiplex gene targeting can generate multiple tagged alleles for a given gene in a single gene targeting experiment, significantly reducing the cost and time required to generate these lines.

While the 3' UTR can contain sequences that affect mRNA stability, *Foxn1 *gene expression and function were not affected in heterozygous or homozygous mice carrying either the Cre or lacZ alleles throughout fetal development to the newborn stage. As the development of lymphoid progenitor cells in the fetal thymus is closely tied to thymic epithelial cell differentiation and function, analysis of thymocyte differentiation can be used as a sensitive and quantitative test for disruption of normal TEC differentiation that would occur if *Foxn1 *gene expression were perturbed. Neither TEC phenotypes, nor thymocyte numbers or differentiation profiles were affected in newborn mice carrying these alleles. These data show that multiplex gene targeting into the 3' UTR of the *Foxn1 *locus can be used as an efficient method for creating multiple alleles to express any gene of interest in all TECs from the earliest stage of thymus organogenesis. By analysis of the published literature, we also identified a number of additional cases where similar 3' UTR targeting strategies have been successful for a wide variety of loci. Our analysis suggests that multiplex gene targeting into the 3' UTR may be a broadly applicable approach.

## Results and discussion

### Multiplex gene targeting at the *Foxn1 *locus

**Figure 1:**
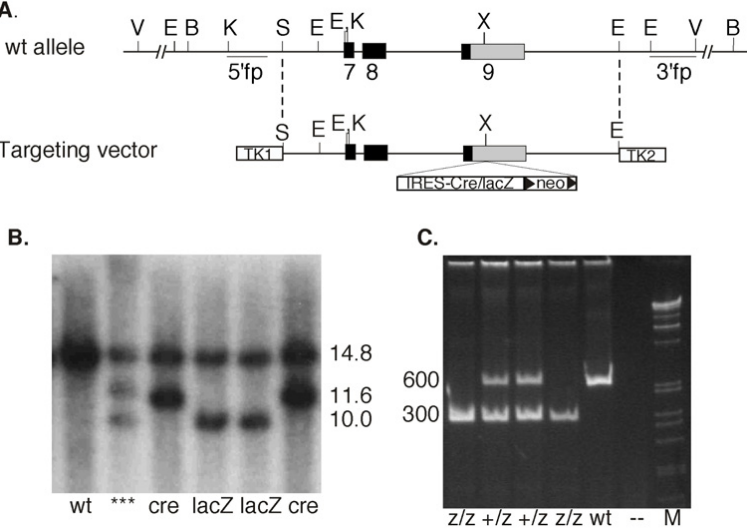
**Multiplex gene targeting at the *Foxn1 *locus**. A. Map of the wild-type (wt) Foxn1 locus and the targeting vectors used for gene targeting. Locations of the 5' and 3' flanking probes (fp) are shown. Exons 7–9 are shown as boxes, coding regions are black and UTR sequences are grey. IRES-Cre or IRES-lacZ cassettes including the floxed or frt neo positive selection cassette were inserted into a unique XhoI site (X) in the 3'UTR in exon 9. The targeting vector homology was flanked by two TKgenes for negative selection. B. Southern blot analysis of ES cell clones. Genomic DNA was digested with EcoRV, and probes with the 5' flanking probe. The wild-type 14.8 kb band is present in all samples. Correctly targeted cell lines with bands corresponding to the two different targeted alleles are present in lanes 3–6. Lane two shows an abberant clone. C. PCR analysis of the lacZ alleles used for genotypeing. The larger, 600 bp band is the wild-type allele, the 300 bp band is the lacZ (z) allele. Marker (M) shown is the Promega 1 kb ladder. V, EcoRV; E, EcoRI; B, BamHI; K, KpnI; S, SspI; X, XhoI.

We built two vectors, both of which were designed to create bicistronic messages expressing the normal *Foxn1 *mRNA followed by either IRES-lacZ or IRES-Cre (Fig. 1A), and utilizing the endogenous polyadenylation sequences after neo cassette deletion. In each vector, either an IRES-lacZ or IRES-Cre cassette was inserted into a unique Xho1 site in exon 9 in the 3' UTR, 257 bases 3' of the termination codon. The IRES sequence used was from ECMV [12]. A mixture of equal amounts of the two linearized vectors was electroporated into either 129Sv or C57BL/6 ES cell lines and subjected to positive-negative selection. 40–45 clones from each ES cell line were screened by Southern blot for presence of a targeting event with a 5' flanking probe, using EcoRV to digest the genomic DNA, which generated different sized bands for targeting events involving each of the two vectors (Fig. 1B).

Results for the 129Sv and C57BL/6 ES cell lines were similar. The overall targeting frequencies were 84% (37/44) in 129SV cells, and 97.5% (40/41) in the C57BL/6 cell line. Frequencies for each vector were such that approximately half of the targeted cell lines were derived from each vector, indicating that the two vectors underwent homologous recombination with equal efficiency. Although the vectors were non-isogenic in the C57BL/6 ES cells, this did not affect either the frequency or fidelity of the targeting events, suggesting that this locus has relatively few polymorphisms between these two strains. Several targeted lines from each vector and each strain were chosen for further analysis. These cell lines were screened with both 5' and 3' flanking probes and with a probe from the *neo*^*r *^selection cassette. All bands were the expected sizes for correct targeting events (data not shown). Subsequently, these alleles were genotyped by PCR (Fig. 1C). We have termed these alleles *Foxn1*^*ex*9*lacZ *^*Foxn1*^*ex*9*cre*^.

### Expression of Cre and lacZ accurately reflects fetal *Foxn1 *gene expression

**Figure 2:**
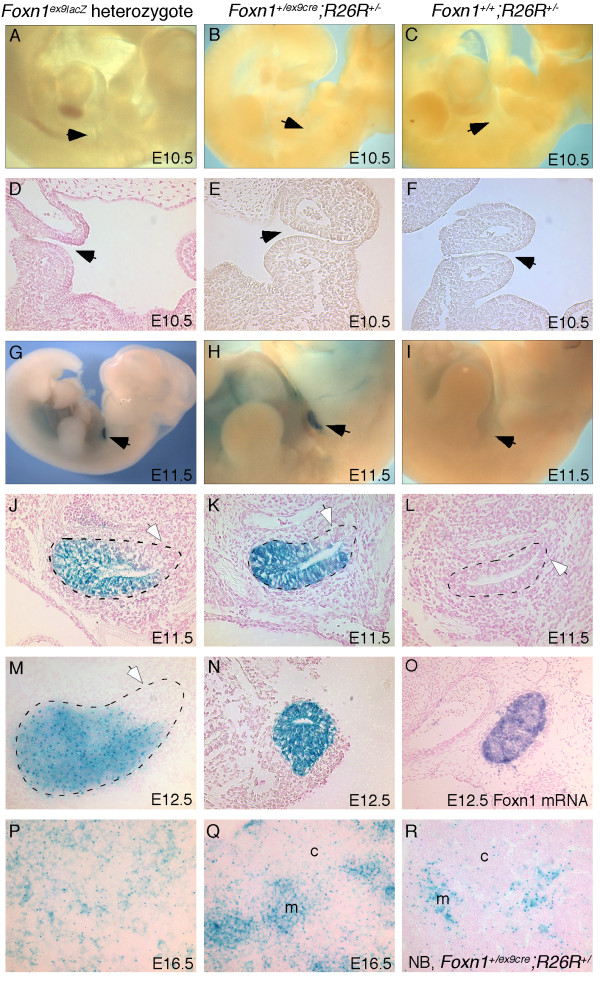
***Foxn1*^*ex*9*lacZ *^and *Foxn1*^*ex*9*cre *^expression from E10.5 to newborn**. Cre expression was assayed using the R26R reporter allele. Panels C, F, I, L show Cre negative control embryos with no expression. Black arrows in (A-I) point to the third pharyngeal pouch. White arrows in (J-L) indicate the *Foxn1*-negative parathyroid region of the primordium. For sagittal sections, ventral is left and anterior is up. For transverse sections, dorsal is up. A, B, Lateral views of E10.5 *Foxn1*^+/*ex*9*lacZ *^(A) and *Foxn1*^+/*ex*9*cre*^;R26R^+/- ^(B) whole embryos, and D, E, coronal sections through E10.5 *Foxn1*^+/*ex*9*lacZ *^(D) and *Foxn1*^+/*ex*9*cre*^;R26R^+/- ^(E) embryos showing no expression in the third pharyngeal pouch. G, H, Lateral views of E11.5 *Foxn1*^+/*ex*9*lacZ *^(G) and *Foxn1*^+/*ex*9*cre*^;R26R^+/- ^(H) embryos showing expression only in the developing thymus primordium. J, K, Sagittal sections through E11.5 *Foxn1*^+/*ex*9*lacZ *^embryo (J) and E11.5 *Foxn1*^+/*ex*9*cre*^;R26R^+/- ^embryo (K) showing expression restricted to the ventral thymus domain of the 3^rd ^pouch-derived organ primordium. M, Sagittal section through E12.5 *Foxn1*^+/*IRESlacZ *^thymus primordium. N, Transverse section through E12.5 *Foxn1*^+/*ex*9*cre*^;R26R^+/- ^thymus primordium. O, Transverse section through E12.5 thymus showing *in situ *hybridization for *Foxn1 *mRNA. P, Section through E16.5 *Foxn1*^+/*ex*9*lacZ *^thymus. Q, R, Sections through E16.5 (Q) and newborn (R) *Foxn1*^+/*ex*9*cre*^;R26R^+/- ^thymi showing more intense staining in the prospective medullary regions. c = cortex, m = medulla.

Bicistronic expression of genes from IRES constructs can result in lower translation efficiencies of the second coding sequence. In the case of Cre, this could result in mosaic Cre-mediated deletion, even with appropriate RNA expression patterns. To test the expression and activity of Cre from the IRES in our construct, we compared the known *Foxn1 *mRNA expression pattern to expression of the *Foxn1*^*ex*9*lacZ *^allele, and to the *Foxn1*^*ex*9*cre *^allele using the R26R reporter strain [13] (*Foxn1*^+/*ex*9*cre*^;*R26R*^+/-^). The timing and localization of initial Cre activity as assayed by the R26R reporter was identical to both lacZ expression from the *Foxn1*^*ex*9*lacZ *^allele and *Foxn1 *mRNA expression. No expression was seen at E10.5 with either allele (Fig. 2A,B,D,E). At E11.5 and E12.5, there was no difference between the *Foxn1*^*ex*9*cre *^allele (Fig. 2H,K,N), the *Foxn1*^*ex*9*lacZ *^allele (Fig. 2G,J,M) and *Foxn1 *mRNA (Fig. 2O and not shown), with expression seen in apparently all epithelial cells of the presumptive thymic rudiment. Heterozygous and homozygous embryos showed similar timing of expression (data not shown). No expression was observed in embryos at E10.5 or E11.5 that did not carry the *Foxn1*^*ex*9*cre *^allele (Fig. 2C,F,I,L). Thus, both alleles faithfully reflected the early expression of *Foxn1*, without affecting the timing or pattern of initial expression from the targeted locus.

We followed the expression of both alleles at E16.5 and newborn stages (Fig. 2P–R and not shown). At E16.5 both alleles showed similar expression patterns, with punctate nuclear staining distributed throughout both the developing cortical and medullary regions (Fig. 2P,Q). Less intense staining in the cytoplasmic regions of the TEC network were also visible, presumably due to inefficient nuclear localization of the lacZ gene product. This effect was particularly obvious in the presumptive medullary regions in the *Foxn1*^+/*ex*9*cre*^;*R26R*^+/- ^thymi (Fig. 2Q,R). This higher level of lacZ activity in medullary regions could be due to a longer half-life for medullary TECs resulting in higher accumulation of lacZ protein, and/or smaller cytoplasmic area for medullary TECs resulting in a relative concentration of the protein.

Using these reporters and at these stages, we did not see any difference between active *Foxn1 *gene expression as indicated by the lacZ allele, versus *Foxn1*-expressing cell lineage, as indicated by the Cre allele (i.e. descendents of all cells who have ever expressed Foxn1). We also did not see a pattern of Foxn1 expression at the newborn stage similar to that recently reported using a Foxn1 antibody [14], in which areas of Foxn1-negative epithelia were seen beginning at the newborn stage. While we cannot rule out the possibility that some TECs may down regulate *Foxn1 *during development, the difference between our results and theirs may be an issue of the differential sensitivity of the reagents used in the two studies.

**Figure 3:**
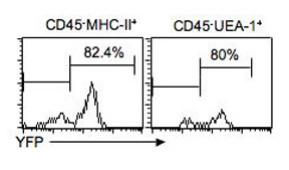
**Analysis of reporter expression in neonatal TECs after Cre recombination**. Five-day old mice carrying both the *Foxn1*^*ex*9*cre *^and *R26YFP *reporter genes were analyzed for the expression of YFP in TEC populations by FACS. Left panel shows the percentage of YFP positive cells in the CD45^-^MHC Class II^+ ^population. Right panel shows the percentage of YFP positive cells in the CD45^-^UEA1^+ ^population.

Ubiquitous expression of Cre in the thymus domain at the initial stages of organogenesis, as indicated by the R262R reporter, should result in permanent deletion in all TECs in a lineage-specific fashion. Consistent with this prediction, we showed in a recent report that when this Cre strain was used to delete the *Foxp3 *gene from TECs, at least 90% of G8.8^+ ^TECs had deleted Foxp3, or were YFP-positive as measured by the R26YFP indicator [15,16]. As a further analysis of the utility of this Cre strain for TEC-specific deletion, we crossed the *Foxn1*^*ex*9*cre *^allele to the R26YFP reporter strain and analyzed the percentage of YFP-positive cells by flow cytometry. By this assay, more than 80% of CD45^-^MHC Class II^+ ^and UEA1^+ ^TECs were YFP^+ ^at 5 days postnatal (Fig. 3). Taken together, these and our previous data show that this Cre strain can direct deletion in a high percentage of TECs.

Our fetal expression data suggest that this is likely to be an underestimate of recombination. Since all epithelial cells at early stages of thymus organogenesis are consistently lacZ positive by the R26R reporter (Figure 2J–O and data not shown), all TECs should be labeled in a lineage-specific fashion throughout the life of the animal. Our analysis of lacZ expression at later fetal stages clearly shows that there are different levels of these reporters in different TEC populations, either due to differential accumulation of the reporter protein due to different cell cycle length, or to actual differences in Rosa26 gene expression (Figure 2Q, R). These differences could result in some cells with lower YFP levels being below the level of detection by flow cytometry.

### Removal of the *neo*^*r *^cassette eliminates ectopic Cre activity

**Figure 4:**
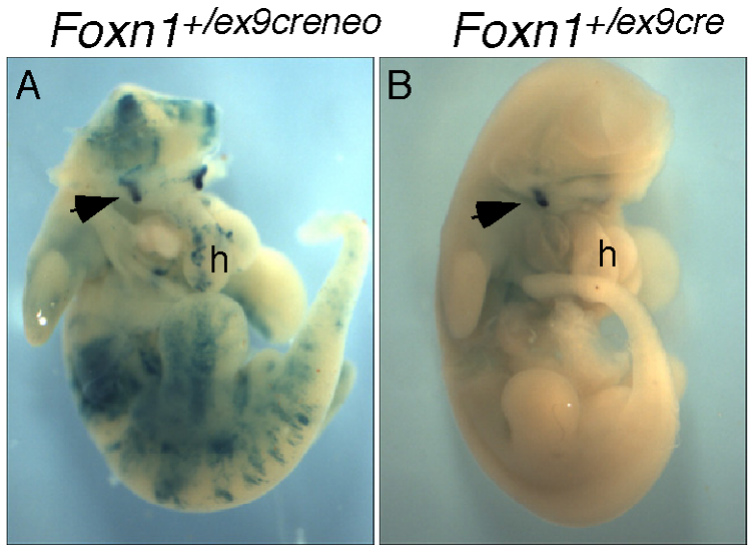
**Effect of *neo*^*r *^deletion on Cre expression**. Ventral views of two E12.5 *Foxn1*^+/*ex*9*cre*^;R26R^+/- ^embryos, x-gal stained to show Cre activity. A, Prior to deletion of the *neo*^*r *^cassette, extensive non-specific staining was seen throughout the embryo. B, Thymus-specific expression after removal of the *neo*^*r *^cassette. The heads have been removed from both embryos to allow visualization of the thymic primordia. Arrows indicate one of the thymic lobes. h = heart.

When we originally tested the *Foxn1*^*ex*9*cre *^strain, the frt-flanked *neo*^*r *^cassette was still present in the locus immediately 3' of the IRES-Cre cassette. Analysis of Cre activity using the R26R reporter strain showed that while all embryos had the expected *Foxn1*-specific Cre activity pattern in the thymus at E12.5, 5/8 embryos tested also showed variable ectopic expression (Fig. 4A). This expression pattern is reminiscent of the strain-dependent and variable ectopic Cre activity previously reported for the Foxg1-Cre mouse strain [17]. In the case of the *Foxn1*^*ex*9*cre *^strain, deletion of the *neo*^*r *^cassette by crossing to the Actin-flp deleter strain [18] resulted in complete loss of ectopic expression with no effect on the *Foxn1*-specific pattern (Fig. 4B). This result provides a dramatic example emphasizing the effect that selectable markers can have on gene expression.

### *Foxn1 *gene expression and function is unaffected in *Foxn1*^*ex*9*lacZ *^and *Foxn1*^*ex*9*cre *^homozygotes

**Figure 5:**
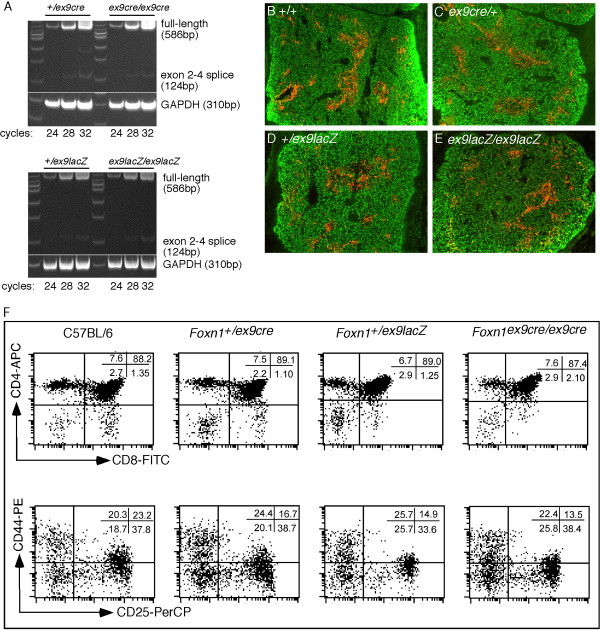
**Phenotypic analysis of mice carrying *Foxn1*^*ex*9*lacZ *^and *Foxn1*^*ex*9*cre *^alleles**. A, Semi-quantitative RT-PCR of *Foxn1 *RNA in thymi from heterozygous and homozygous *Foxn1*^*ex*9*cre *^or *Foxn1*^*ex*9*lacZ *^newborn mice. Both the 586 bp Foxn1 full-length product and the 124 bp exon 2–4 spliced product are first seen at 24 cycles in all genotypes. B-E, Keratin 5 and keratin 8 expression in the E16.5 thymus. Transverse sections through E16.5 thymic lobes from (B) wild-type, (C) *Foxn1*^+/*ex*9*cre*^, (D) *Foxn1*^+/*ex*9*lacZ *^and (E) *Foxn1*^*ex*9*lacZ*/*ex*9*lacZ *^embryos. Keratin 8 is shown in green and marks cortical epithelial cells, while keratin 5 marks medullary epithelial cells and is shown in red. F, FACS analysis of thymocytes from *Foxn1*^*ex*9*lacZ *^and *Foxn1*^*ex*9*cre *^mice. Thymocytes from newborn mice were stained with anti-CD4-APC, anti-CD8-FITC, anti-CD44-PE, and anti-CD25-Biotin followed by Streptavidin-PerCP. The upper panels show expression of CD4 and CD8 on total thymocytes. The lower panels show expression of CD44 and CD25 on the gated CD4^- ^CD8^- ^subpopulation.

A common problem with mouse strains carrying either marker genes or Cre inserted into the coding sequence of the target locus ('knock-in/knock-out' strains) is the disruption of and loss of function for the targeted locus. While this is convenient for the simultaneous analysis of gene expression and mutant phenotype, it can cause unexpected or undesirable phenotypic complications when crossed with other mutations due to genetic interactions between the two genes. In addition a number of mouse genes are haploinsufficient suggesting that the knockin/knockout strategy would often lead to phenotypes in the driver strain itself. This could confound the analysis of mice containing the driver locus in combination with other mutant allele(s) at other loci. The 3' UTR strategy we have employed should greatly minimize the potential for phenotypic effects due to the driver locus itself, as long as the insertion does not affect the expression of the targeted locus. Semi-quantitative RT-PCR showed similar levels of *Foxn1 *RNA in newborn thymi from heterozygous and homozygous *Foxn1*^*ex*9*cre *^or *Foxn1*^*ex*9*lacZ *^(Fig. 5A) mice. These results confirmed that the insertion into the 3' UTR did not affect steady-state *Foxn1 *mRNA levels.

We further performed a phenotypic analysis of fetal TEC differentiation and thymocyte development in these two mouse strains. TEC differentiation and initial medullary formation appeared normal as measured by immunostaining for Keratin 8 (K8) and Keratin 5 (K5) expression [19]. At E16.5 the normal thymus contains small areas of prospective medullary epithelium, as shown by the presence of clusters of K8^*lo*^K5^+ ^or K8^-^K5^+ ^cells (Fig. 5B). This staining pattern was seen in thymi from E16.5 *Foxn1*^+/*ex*9*cre *^and *Foxn1*^*ex*9*lacZ *^heterozygous and homozygous embryos (Fig. 5C–E).

We used analysis of thymocyte differentiation as a sensitive and quantitative test for disruption of normal TEC differentiation that would occur if *Foxn1 *gene expression were perturbed (Fig. 5F). *Foxn1*^+/*nu *^heterozygotes have a mild haploinsufficiency phenotype, with lower total thymocyte numbers but normal thymocyte differentiation patterns ([20]; our unpublished observations). Thymi from both *Foxn1*^*ex*9*cre *^and *Foxn1*^*ex*9*lacZ *^heterozygous and homozygous newborns had normal thymocyte development, as measured by thymocyte cell number and differentiation profiles when compared to wild type newborn mice (Fig. 5F and not shown). Similar results were obtained when the *Foxn1*^*ex*9*cre *^and *Foxn1*^*ex*9*lacZ *^alleles were crossed to the *Foxn1 *null nude allele (data not shown). These results demonstrated that both heterozygotes and homozygotes for the *Foxn1*^*ex*9*cre *^and *Foxn1*^*ex*9*lacZ *^alleles had normally functional thymi at this stage, further indicating that the 3' UTR insertions did not affect *Foxn1 *expression or function.

### Multiplex gene targeting and high-efficiency loci

The current results provide evidence that any loci that undergo high efficiency gene targeting in ES cells are candidates for multiplex gene targeting to generate multiple alleles in one step. These alleles could be part of an allelic series for functional analysis or driver loci designed for the expression of exogenous genes in specific spatial or temporal patterns. To determine whether this strategy could be applied to a large number of genes we performed literature mining to document published gene targeting frequencies. It is not possible to do a comprehensive analysis of the entire gene targeting literature since most investigators stopped including targeting frequencies in their papers in the mid-1990s. However, our search suggests that there are many genes available that can be easily used to generate multiple driver alleles to express recombinases and/or marker genes in specific temporal and spatial patterns.

Overall, we were surprised at the total number of very easily targeted genes. A recent study found that a number of genes are expressed at a high enough level in ES cells to enable the use of promoterless selection cassettes in the targeting vector (gene traps), thereby greatly enhancing targeting frequencies [21]. In a set of 29 loci, 17 had targeting frequencies of 45%–95% [21]. Our search of the "classical" gene targeting literature identified additional loci that undergo gene targeting at a relatively high frequency. We focused on genes for which no gene trap ES cell lines are listed on the UCSC genome browser (Feb 2006 assembly) suggesting that a promoterless targeting strategy could not be applied in these cases. We found 6 genes in addition to *Foxn1 *that exhibit high targeting efficiency with 5 of these loci (*Hoxa1, Nkx2.5, Gsc, Cdx1*, and *Nos3*) have targeting efficiencies of 20%–40% while HPRT can be targeted at 100% efficiency [22-28]. In addition to these loci, we have identified an additional locus in our own work, the *Viaat *gene, that also undergoes gene targeting at a high efficiency (40%) after positive-negative selection (W.-J. Oh and B.G. Condie manuscript in preparation). We have also recently obtained similar results for multiplex gene targeting at the Rosa26 locus, which has a targeting frequency of 60–80% in our lab (Z. Liu and N.R. Manley, unpublished). As greater than 90% of the targeted cell lines in both the Rosa26 and *Foxn1 *loci showed correct allele structure, this approach could be of even more utility in the case of genes with lower frequencies, as it would allow screening for multiple rare targeting events simultaneously in a single experiment. Given that a number of genes appear to undergo gene targeting at a high efficiency (greater than 20%), knockin approaches at many loci may be easier than initially appreciated.

### The 3'UTR as a location for driver and marker insertion

In the current study we integrated the Cre recombinase and lacZ genes into the 3' UTR of the *Foxn1 *gene. We chose this strategy to minimize the possibility that the insertion will disrupt *Foxn1 *function, a particular concern because of our evidence showing dosage sensitivity for the *Foxn1 *gene. However, as the 3'UTR is documented to be the location of sequences that affect the stability and translation of mRNAs, it is unclear whether this strategy could be widely applicable to the generation of driver loci. To gain an indication of how widely applicable this approach can be, we performed an extensive analysis of published driver strains to see how often this knockin strategy has been used to generate Cre driver mouse strains. We made particular note of any documented effects of 3' UTR insertions on the function of the driver (host) gene. While this strategy does not identify cases in which a similar approach failed and were therefore not published, it can provide an indication of whether our results with the *Foxn1 *locus represent an isolated incident or are similar to results obtained at other loci.

Our search uncovered 20 published 3' UTR IRES-Cre knockin mouse strains (Table 1). In 10 of the mouse strains, the authors had tested the effect of the 3' UTR knockin on the function of the driver locus by genetic analysis. In 9 out of the 10 cases, the normal expression and function of the driver locus was not affected by the 3'UTR knockin (Table 1). It is noteworthy that this approach enabled the construction of a Sox9-Cre knockin strain that could not have been generated using a knockin/knockout approach, as null mutants for *Sox9 *are heterozygous lethal [29]. We also found that the 3' UTR knockin strategy resulted in the generation of Cre strains that faithfully expressed Cre recombinase activity in the same pattern as the driver locus (Table 1).

**Table 1:** Published 3'UTR IRES-Cre mouse strains

**GENE**	**Cre expression same as driver gene?**	**Phenotype of IRES-Cre homozygote**	**Reference**
*Gdf7*	Yes	ND	[35]
*Emx1*	Yes	Wild type	[36]
*Nkx2-5*	Yes	Hypomorphic?	[26]
*Pvalb (PV)*	Yes	ND	[37]
*Hoxb1*	ND	Wild type	[38]
*Hoxa3*	Yes	ND	[39]
*AP2α (Tcfap2a)*	Yes	ND	[39]
*Sox9*	ND	Wild type*	[29]
*SA*	Yes	Wild type	[40, 41]
*Myf-6*	Yes	Wild type	[41]
*Pax7*	Yes	Wild type	[42]
*(Olfr1507) (MOR28)*	Yes	ND	[43]
*Olfr 151 (M71)*	Yes	ND	[44]
*Slc6a3 (DAT)*	Yes	ND	[45]
*Wnt3a*	Yes	Wild type	[46]
*OMP*	Yes	ND	[47]
*ObRb*	Yes	ND	[48]
*Ntrk3*	Yes	Wild type	[49]
*TH*	Yes	Wild type	[50]
*Rgs9-2*	ND	ND	[51]

*Heterozygous *Sox9 *null mutants die shortly after birth.

ND = not done

Our results with *Foxn1 *3' UTR and the previously published examples of highly successful 3' UTR knockin alleles strongly supports the use of this strategy for the generation of driver alleles. It is important to emphasize that our study and most if not all of the published Cre drivers made 3'UTR driver alleles without regard to the location of possible 3'UTR regulatory sequences. As many of these consensus sequences are now well known [30,31], combining a simple search for potential elements with the 3'UTR insertion strategy should significantly improve the likelihood of success for this approach. The ability to introduce sequences into a mouse locus without disrupting the normal function of that locus minimizes the chances of possible confounding genetic interactions when these alleles are combined with mutant alleles at other loci.

## Conclusion

The current results show that by combining the high targeting efficiency at the *Foxn1 *locus with a 3' UTR insertion strategy, we have generated two highly useful *Foxn1 *alleles. We have consistently obtained very high gene targeting efficiencies at two different locations within the *Foxn1 *locus [11](current report), as have other investigators [5]. This extremely high gene targeting efficiency makes it very easy to modify the *Foxn1 *locus by homologous recombination in embryonic stem cells. The ease of gene targeting will facilitate future analysis of Foxn1 functions *in vivo*. Insertion of the IRES cassette in the *Foxn1 *3' UTR did not affect either the expression pattern or level of *Foxn1*, yielding Cre or lacZ expression and activity in a pattern identical to that of the *Foxn1 *gene. Flow cytometric analysis of early postnatal thymus confirmed that the Cre activity resulted in deletion in a high percentage of TECs. Functional analysis of the fetal thymus showed a completely normal wild-type phenotype, further demonstrating that the 3' UTR insertion site did not affect gene expression. These results indicate that these alleles can be used in combination with other mutations in other genes with little likelihood of confounding genetic interactions due to dosage effects of *Foxn1*. Therefore, the alleles generated in the current study provide key reagents for the in vivo analysis of TEC differentiation and function.

These results have significant implications for the design and construction of driver alleles by knockin strategies. Our results show that multiplex gene targeting is an effective and efficient method to generate at least two independent alleles for a given locus in a single gene targeting experiment. Using this approach can significantly reduce the cost of generating an allelic series for a given gene, as long as the different modifications are made at the identical location within the gene. In addition, while the 3' UTR is a common location for sequences that influence mRNA stability, analysis of the *Foxn1 *Cre and lacZ 3' UTR knockin strains and the successes of other investigators in generating Cre driver alleles at multiple loci by similar 3' UTR knockin strategies provide evidence that this approach may be generally applicable for the generation of driver lines without additional gene dosage complications. These results therefore suggest that this approach represents a broadly useful strategy that may be applied to many loci.

## Methods

### Targeting vector construction

Targeting vectors were constructed using standard cloning techniques. The unique Xho1 insertion site was flanked by 3.5 kb of 129Sv genomic DNA on the 5' side and 7 kb on the 3' side. Inserts contained either the IRES-Cre or IRES-lacZ cassette, followed by an frt-PGK-neo-polyA-frt cassette for positive selection. A dual-TK vector was used for negative selection [32,33]. A mixture of equal amounts of the two linearized vectors was electroporated into either 129Sv or C57BL/6 ES cell lines and subjected to positive-negative selection. 40–45 clones from each ES cell line were screened by Southern blot for presence of a targeting event with a 5' flanking probe, using EcoRV to digest the genomic DNA, which generated different sized bands for targeting events involving each of the two vectors (Fig. 1B). Correctly targeted cell lines were further screened with additional enzymes and with a 3'flanking probe to ensure correct allele structure.

### Electroporation and ES cell culture

ES cell culture and generation of mouse strains was performed in the Medical College of Georgia Embryonic Stem Cell and Transgenic core facility.

### X-gal staining

For E10.5 and E11.5 embryos, x-gal staining was done on whole embryos, which were sectioned and counterstained with nuclear fast red, as previously described [5]. X-gal staining of E12.5 and E16.5 embryos was done on frozen sections. Whole embryos or isolated thymic lobes were embedded in optimum cutting temperature compound (Sakura) and frozen on dry ice. 10 μm sections were cut and stained using the same protocol as for whole embryos.

### Semi-quantitative RT-PCR

Isolation of RNA from newborn thymi and semi-quantitative RT-PCR were performed as previously described [11]. *Foxn1 *exon 2–4 full-length product = 586 bp. Exon 3-deleted product = 124 bp. GAPDH primers were used as standard (310 bp product).

### Immunofluorescence

Whole E16.5 embryos were 'flash frozen' in liquid nitrogen and 10 μm sections were cut and stained as previously described [11]. The keratin 5 polyclonal primary antibody (Covance) was used with a Texas Red-donkey anti-rabbit IgG secondary antibody. The keratin 8 primary antibody (Troma-1 supernatant) was used with a FITC-donkey anti-rat IgG secondary antibody.

### Flow cytometry

Thymocyte suspensions were prepared from thymi from newborn C57BL/6, *Foxn1*^*ex*9*cre *^homozygous, *Foxn1*^*ex*9*lacZ *^homozygous and *Foxn1*^*ex*9*lacZ *^heterozygous mice. The red blood cells were lysed and total cell numbers were counted. 1 × 10^6 ^thymocytes were stained with the following monoclonal antibodies conjugated to PE, FITC, APC directly, or Biotin-labeled monoclonal antibodies, followed by streptavidin-PerCP: anti-CD4 (RM4-5), anti-CD8a (53-6.7), anti-CD44 (IM7) or anti-CD25 (7D4) (BD Pharmingen, San Diego, CA). Anti-CD16/32 (2.4G2) (BD Pharmingen) and normal rat serum were used to block FC-receptors before staining. Four-color immunofluorescence analysis was performed using a FACSCalibur system. The data were analyzed using CellQuest software (Becton Dickson, Franklin Lake, NJ).

### Thymic stromal cell isolation and analysis

Thymi were removed from day 5 *R26YFP*^+/*tg*^;*Foxn1Cre*^+/- ^mice and individually put into a 70 μm strainer in a Petri dish containing 15 ml of 5% FBS + RPMI-1640 medium on ice. Each thymus was cut into 1 mm^3 ^size pieces with a pair of sharp scissors and the thymic fragments were washed three times to remove the majority of thymocytes. After washing with RPMI-1640 medium once, the thymic fragments were transferred into 8 ml RPMI-1640 containing 1 mg/ml collagenase IV (Sigma) and 5 ug/ml DNase I (Invitrogen) and then incubated at 37°C for 30 minutes. After adding EDTA to 2 mM final concentration, digested thymic cells were layered on 4 ml of 12%Opti-Perp density gradient solution and centrifuged at 2000 rpm for 20 minutes at 10°C. Thymic stromal cells were collected and enriched from interface cells, washed and then resuspended in 2% FBS +PBS + 2 mM EDTA buffer for FACS analysis. Anti-CD45 APC, MHC-II PE and UEA-1 Biotin (Vector Laboratories Inc.) following Avidin-PerCP were used. Data was collected on a FACSCalibur and the data were analyzed using CellQuest software (Becton Dickson).

### Data mining from published literature and online sources

To locate publications reporting targeting frequencies we searched PubMed with the term "gene targeting" and examined publications from 1987–1996 that reported the generation of gene targeted alleles. Only papers available online were examined. To locate publications describing the generation of Cre driver alleles via the knockin of an IRES Cre into the 3' UTR we performed searches using Google, PubMed and Chilibot [34]. We also searched the Mouse Genome Informatics database (MGI, ). We performed the Google, Pubmed and MGI searches with the query "ires cre" and a Chilibot search with the keyword pair "ires" and "cre".

## Authors' contributions

JG performed the analysis of Cre activity and lacZ expression, and participated in coordination of the study and drafting the manuscript. SX performed the quantitative analysis of YFP expression in TECs, and with DMS performed the analysis of thymocyte development. BH performed the immunofluorescence analysis and participated in gene expression analysis. DMS performed the RT-PCR analysis. SPN generated the targeting vectors and identified and confirmed the targeted cell lines and mouse strains. BGC conceived and performed the meta-analysis of the 3'UTR gene targeting literature, and participated in data analysis and drafting the manuscript. NRM conceived and designed the study, and participated in its coordination and the interpretation of data, and drafted the manuscript. All authors read and approved the final manuscript.
